# Significance of Lipopolysaccharides in Gastric Cancer and Their Potential as a Biomarker for Nivolumab Sensitivity

**DOI:** 10.3390/ijms241411790

**Published:** 2023-07-22

**Authors:** Nobuhiro Nakazawa, Takehiko Yokobori, Makoto Sohda, Nobuhiro Hosoi, Takayoshi Watanabe, Yuki Shimoda, Munenori Ide, Akihiko Sano, Makoto Sakai, Bilguun Erkhem-Ochir, Hiroomi Ogawa, Ken Shirabe, Hiroshi Saeki

**Affiliations:** 1 Department of General Surgical Science, Graduate School of Medicine, Gunma University, Maebashi 371-8510, Japan; nakazawa75@yahoo.co.jp (N.N.); m11201085@gunma-u.ac.jp (N.H.); twatanabe10001@gmail.com (T.W.); ak_sano@gunma-u.ac.jp (A.S.); maksakai@gunma-u.ac.jp (M.S.); hiroomio@gunma-u.ac.jp (H.O.); kshirabe@gunma-u.ac.jp (K.S.); h-saeki@gunma-u.ac.jp (H.S.); 2 Division of Integrated Oncology Research, Gunma University, Initiative for Advanced Research (GIAR), Maebashi 371-8511, Japan; bilguunerkhemochir@gmail.com; 3 Department of Diagnostic Pathology, Gunma University Graduate School of Medicine, Gunma University, 3-39-22 Showa-machi, Maebashi 371-8511, Japan; yshimoda@gunma-u.ac.jp (Y.S.); idemsurvive@gmail.com (M.I.)

**Keywords:** epithelial–mesenchymal transition (EMT), gastric cancer, lipopolysaccharides (LPS), nivolumab, TGF-β

## Abstract

Lipopolysaccharides are a type of polysaccharide mainly present in the bacterial outer membrane of Gram-negative bacteria. Recent studies have revealed that lipopolysaccharides contribute to the immune response of the host by functioning as a cancer antigen. We retrospectively recruited 198 patients with gastric cancer who underwent surgery. The presence of lipopolysaccharides was determined using immunohistochemical staining, with the intensity score indicating positivity. The relationship between lipopolysaccharides and CD8, PD-L1, TGFBI (a representative downstream gene of TGF-β signaling), wnt3a, and E-cadherin (epithelial–mesenchymal transition marker) was also investigated. Thereafter, we identified 20 patients with advanced gastric cancer receiving nivolumab and investigated the relationship between lipopolysaccharides and nivolumab sensitivity. After staining for lipopolysaccharides in the nucleus of cancer cells, 150 negative (75.8%) and 48 positive cases (24.2%) were found. The lipopolysaccharide-positive group showed increased cancer stromal TGFBI expression (*p* < 0.0001) and PD-L1 expression in cancer cells (*p* = 0.0029). Lipopolysaccharide positivity was significantly correlated with increased wnt3a signaling (*p* = 0.0028) and decreased E-cadherin expression (*p* = 0.0055); however, no significant correlation was found between lipopolysaccharide expression and overall survival rate (*p* = 0.71). In contrast, high TGFBI expression in the presence of LPS was associated with a worse prognosis than that in the absence of LPS (*p* = 0.049). Among cases receiving nivolumab, the lipopolysaccharide-negative and -positive groups had disease control rates of 66.7% and 11.8%, respectively (*p* = 0.088). Lipopolysaccharide positivity was associated with wnt3a, TGF-β signaling, and epithelial–mesenchymal transition and was considered to tend to promote therapeutic resistance to nivolumab.

## 1. Introduction

Gastric cancer (GC), one of the most common digestive cancers, is the third leading cause of cancer-related death worldwide [1]. The clinical outcomes of patients with GC, particularly overall survival (OS), has gradually improved following the introduction of chemotherapy and immune checkpoint inhibitors (ICIs). In fact, ATTRACTION-2, the first phase 3 study on patients with GC receiving two or more chemotherapy regimens, showed that the anti-Programmed death receptor-1 (PD-1) antibody nivolumab improved overall survival (OS) [2]. Furthermore, the CheckMate 649 study recommended nivolumab plus chemotherapy as the primary treatment for GC [3]. However, patients with GC have shown unsatisfactory response rates to ICIs. Concerns regarding the impact of expensive ICIs on the health care economy have also emerged. As such, a robust biomarker is needed to more effectively assess the positive clinical therapeutic effects of nivolumab in patients with GC.

The cell wall of Gram-negative bacteria is composed of LPS. Nejman et al. showed that intratumor bacteria were present in cancer cells and were mostly intracellular. They also showed that bacterial lipopolysaccharides (LPS) were present in tumor cells and that intratumor bacteria could affect responses to immunotherapy. They conducted a comprehensive analysis of the microbiome, and they clarified the intratumor bacteria were mostly intracellular and were present in both the nucleus of cancer cells and in immune cells by electron microscopy [4]. Recent studies have revealed that LPS contributes to the immune response of the host by functioning as a cancer antigen. Furthermore, in their examination of melanoma, they demonstrated that the bacteria were present within the cancer cells, not in the extracellular microenvironment. They found that the intracellular flora was very similar in many metastases of the same patient, triggering an immune response [5]. Reports have shown that LPS were associated with the development of GC by regulating cell proliferation, autophagy, and epithelial–mesenchymal transition (EMT) [6,7,8]. In particular, Helicobacter pylori LPS have been found to activate both the Nuclear Factor-κB (NFkB) and signal transducers and activate the transcription 3 (STAT3) signaling pathways [9,10,11]. NFkB induces Programmed cell Death ligand 1 (PD-L1) expression and EMT by activating transforming growth factor β (TGF-β) signaling [12].

TGF-β plays a critical role in EMT, which causes therapeutic resistance to ICIs [13,14]. In particular, TGF-β activates a pathway mediated by small mother against decapentaplegic (SMAD) proteins that induces the downstream activation of EMT, including N-cadherin and vimentin, via Snail-1, Twist, and ZEB1 [15,16]. However, the appropriate method for evaluating TGF-β signaling in patients with cancer remains elusive, given the genetic variations in TGF-β pathway genes across several cancers [17,18]. Our previous report showed that high stromal transforming growth factor-beta-induced protein (TGFBI) in lung cancer was associated with poor prognosis and therapeutic resistance to ICIs [19]. TGFBI had initially been described as a protein strongly induced by TGF-β and one of the representative downstream genes in TGF-β signaling [20]. However, few studies have addressed the relationship between LPS and TGFBI, as well as the therapeutic effects of the anti-PD-1 antibody nivolumab in GC.

The current study, therefore, aimed to clarify the significance of LPS in patients with GC. Moreover, we investigated whether LPS could be a biomarker for predicting treatment resistance in patients receiving nivolumab for GC.

## 2. Results

### 2.1. Presence of LPS in GC Surgical Tissues

**Figure 1:**
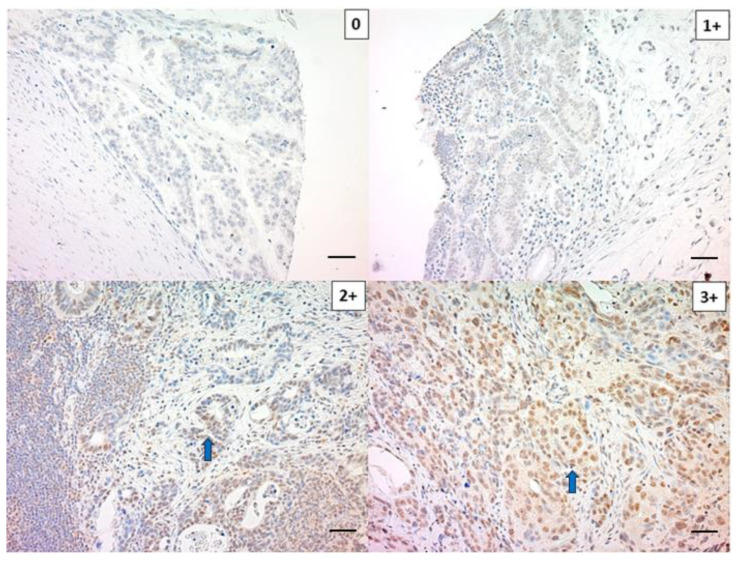
Representative images of the immunohistochemical staining of lipopolysaccharides (LPS). The scale bar represents 20 μm. 0; no staining, 1+; weak staining, 2+; moderate staining, 3+; strong staining. Blue arrow indicates the presence of LPS.

LPS were stained in the nucleus of cancer cells (Figure 1), with our results showing 150 (75.8%) and 48 (24.2%) negative and positive cases, respectively.

### 2.2. Relationship between LPS and Clinicopathological Factors of Patients with GC

**Figure 2:**
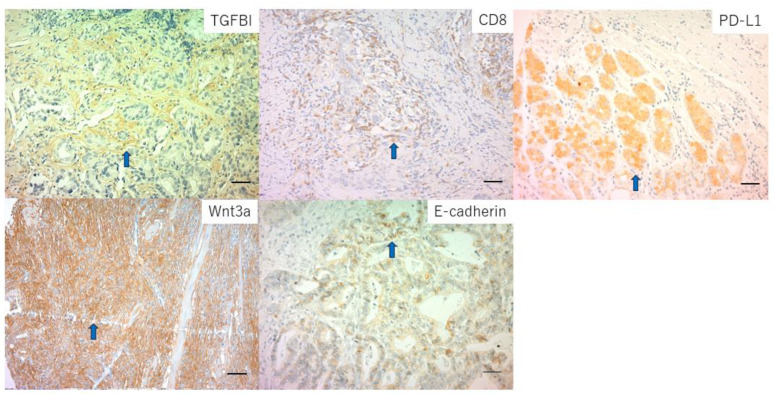
Representative images of the immunohistochemical staining of transforming growth factor-beta-induced protein (TGFBI), CD8, Programmed cell Death ligand 1 (PD-L1), Wnt3a, and E-cadherin. The scale bar represents 20 μm. Blue arow indicates stain positive cells.

We showed the representative images, such as TGFBI, CD8, PD-L1, Wnt3a, and E-cadherin, in Figure 2. The results for the relationship between LPS and clinicopathological factors are summarized in Table 1. Accordingly, no significant difference in age, sex, tumor size, depth, differentiation, presence of lymph node metastasis, and stage were observed between positive and negative cases. The LPS-positive group showed significantly greater cancer stromal TGFBI expression (*p* < 0.0001), more HER2-negative cases (*p* = 0.012), greater PD-L1 expression (*p* = 0.0029), increased wnt3a signaling (*p* = 0.0028) and lower E-cadherin expression (*p* = 0.0055) (EMT marker) than the LPS-positive group.

### 2.3. Kaplan–Meier Curve for Overall Survival According to the Presence of LPS in Surgical Cases with GC

**Figure 3:**
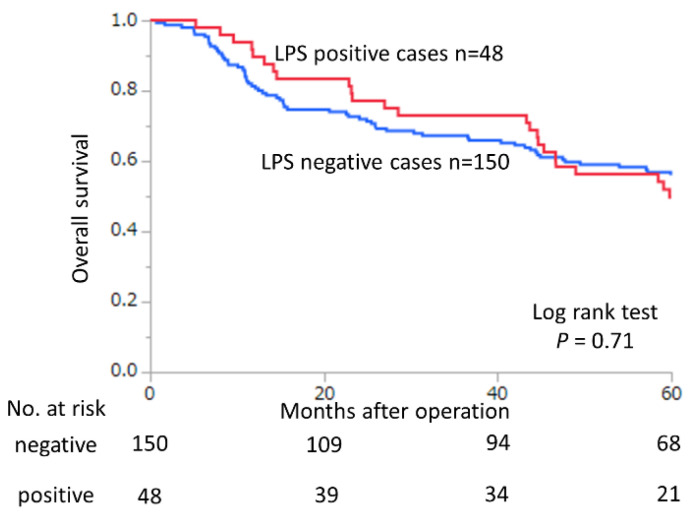
Kaplan–Meier analysis of 5-year overall survival in relation to lipopolysaccharides (LPS).

Figure 3 presents the Kaplan–Meier curve for overall survival according to the presence of LPS in surgical cases with GC. Accordingly, the presence of LPS was not significantly associated with overall survival (*p* = 0.71).

### 2.4. Kaplan–Meier Curve for Overall Survival with TGFBI Expression and Prognosis According to the Presence or Absence of LPS in Surgical Cases with GC

**Figure 4:**
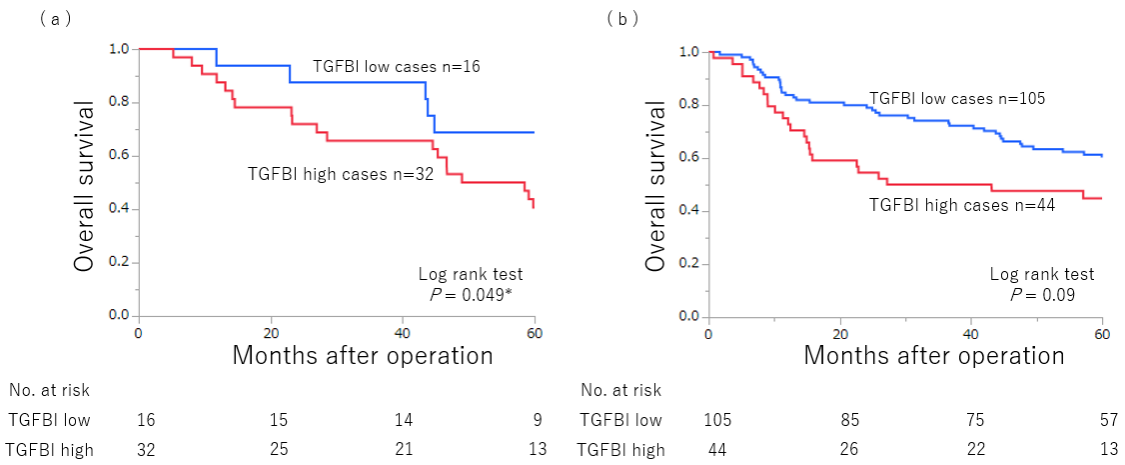
Kaplan–Meier analysis of 5-year overall survival in relation to transforming growth factor-beta-induced protein (TGFBI) expression and prognosis according to the presence or absence of lipopolysaccharides (LPS). (**a**) Presence of LPS, and (**b**) absence of LPS. * *p* < 0.05.

Figure 4 also presents the Kaplan–Meier curve for overall survival with TGFBI expression and prognosis according to the presence or absence of LPS in surgical cases with GC. High TGFBI expression in the presence of LPS was associated with a worse prognosis than that in the absence of LPS (*p* = 0.049). On the other hand, the absence of LPS showed a trend toward poor prognosis in the high TGFBI expression, but the difference was not statistically significant.

### 2.5. Relationship between LPS and Nivolumab Sensitivity

Table 2 outlines the results for the relationship between LPS and nivolumab sensitivity. Notably, the LPS-negative and -positive groups showed disease control rates of 66.7% and 11.8%, respectively (*p* = 0.088). The results showed a correlation trend between LPS and nivolumab sensitivity, although the difference was not statistically significant.

## 3. Discussion

The current study showed that LPS in GC cells were associated with enhanced TGF-β signaling, wnt3a signaling, PD-L1 expression, and EMT. Although the presence of LPS did not contribute to the prognosis of GC, high TGFBI expression in the presence of LPS was associated with a worse prognosis than that in the absence of LPS (*p* = 0.049). On the other hand, the LPS-negative and -positive groups showed disease control rates of 66.7% and 11.8%, respectively (*p* = 0.088). The results showed a correlation trend between LPS and nivolumab sensitivity, although the difference was not statistically significant.

A previous report revealed that intratumor bacteria were present in both cancer and immune cells and were mostly intracellular. Evidence has also shown that intratumor bacteria or their predicted functions were correlated with tumor types and subtypes, patients’ smoking statuses, and responses to immunotherapy [4]. Moreover, recent studies have revealed that LPS contributes to the immune response of the host by functioning as a cancer antigen [5]. In line with this, our study showed that LPS contributed to therapeutic resistance in patients receiving nivolumab for GC.

**Figure 5:**
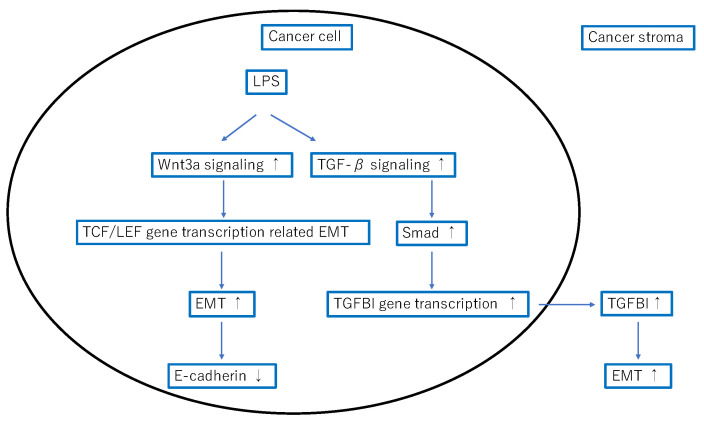
Hypothesis of lipopolysaccharides (LPS) triggered epithelial–mesenchymal transition (EMT) by activating Wnt3a and TGF-β signaling.

Epithelial cells can acquire a mesenchymal phenotype through a process called EMT. More specifically, EMT can be described as a process of cell plasticity in which epithelial cells acquire mesenchymal characteristics, such as fibroblast-like morphology, increased motility, and increased expression of mesenchymal markers such as vimentin while simultaneously decreasing cell-to-cell contact, E-cadherin and claudin expression, and other epithelial characteristics [21]. Cytotoxin-associated gene A (CagA) is the major virulence factor and oncoprotein of H. pylori [22]. Previous reports have shown that CagA promoted EMT by reducing GSK-3 activity [23] and downregulating programmed cell death protein 4 [24]. The current study showed that LPS, a constituent molecule of Gram-negative bacteria, was present in cancer cells, suggesting a new possibility for inducing EMT. We also showed that the activation of wnt3a signaling may downregulate E-cadherin expression and induce EMT during these processes (Figure 5).

TGF-β signaling is considerably complex in addition to being time- and context-dependent, with different signaling processes occurring in cancerous and noncancerous areas [25]. Hence, evaluating TGF-β signaling is quite challenging. TGFBI is a representative downstream gene of TGF-β signaling and a protein whose expression is induced downstream of SMAD [20]. In other words, TGFBI expression can be a good indicator of TGF-β signaling activation. Furthermore, one study showed that TGFBI expression is highly induced in the process of EMT [26]. LPS induced IL-6 and TGF-β1 secretion in esophageal cancer cell lines, and E-cadherin was significantly decreased after LPS treatment, promoting EMT [27]. Interestingly, the current study showed that cancer stromal TGFBI expression was strongly correlated with the presence of LPS in cancer cells. This indicated that bacterial-derived LPS activated TGF-β signaling, suggesting that LPS regulation may be useful for regulating TGF-β signaling and, consequently, EMT (Figure 5).

Our previous study reported that cancer stromal TGFBI expression was significantly associated with therapeutic resistance in patients receiving nivolumab for lung cancer [18]. Similarly, the present study found that the presence of LPS was significantly correlated with cancer stromal TGFBI expression, which may have contributed to therapeutic resistance in patients with GC using nivolumab. Furthermore, reports of increased EMT and resistance to ICI treatment have been observed in other cancers [28,29]. Although the presence of LPS was positively correlated with PD-L1 expression in cancer cells in the present study, the aforementioned EMT findings suggested that the presence of LPS may have caused treatment resistance in patients receiving nivolumab for GC.

V Gopalakrishnan et al., who studied the oral and gut microbiota of patients with melanoma receiving anti-PD-1 immunotherapy, reported significant differences in the diversity and composition of patient gut bacteria between responders and non-responders. Immune profiling also suggested enhanced systemic and antitumor immunity in reactive patients with favorable gut microbiota and in sterile mice transplanted with feces from reactive patients [30]. Given that H. pylori infections have been suggested to cause GC, we would like to examine the history of H. pylori eradication and sensitivity to ICIs in the future.

Our study has several limitations worth noting. First, this is a single-center, retrospective study with a small sample size. In particular, the GC samples treated by nivolumab might not be enough to indicate strong evidence of LPS as a promising biomarker for ICIs treatment. And, almost-surgical or biopsy specimens in this study were not obtained just before nivolumab. Further prospective studies are needed to clarify the importance of LPS evaluation as an ICI sensitivity marker for GC patients in the clinic. We also need to evaluate the significance of LPS with the first line of GC chemotherapy because ICIs are used in first-line chemotherapy. Second, no in vitro or in vivo studies were conducted herein. Third, this study did not examine the association between LPS and H. pylori. In the future, we would like to use surgical specimens to investigate the relationship between 16srDNA and LPS at cancer sites. Finally, lymphocytes were also stained for LPS in immunostaining. The significance of this staining has not been examined.

In conclusion, the current study found that LPS was associated with wnt3a, TGF-β signaling, and EMT, and its expression affected therapeutic resistance to nivolumab in patients with GC.

## 4. Materials and Methods

### 4.1. Patients

This study included 198 patients who underwent potentially curative surgery for GC at the Department of General Surgical Science, Gunma University Hospital between 1996 and 2006. In this study, we performed tissue microarray analysis. Preoperatively treated cases and endoscopic mucosal resection cases were excluded, and multiple blocks containing preinvasive sites were collected and subjected to tissue microarray. Clinicopathological factors, such as age, sex, tumor size, depth, differentiation, presence of lymph node metastasis, stage, and HER2 status, were determined. Furthermore, we analyzed 20 patients receiving nivolumab for postoperative recurrent or unresectable advanced GC from 2017 to 2021 in our department. Nivolumab was used after third-line treatment in all cases. Among such cases, the response to nivolumab was evaluated using the Response Evaluation Criteria in Solid Tumors version 1.1 [31]. The resected specimens and pretreatment biopsy specimens were included in nivolumab sensitivity consideration. Our study was approved by the institutional review board of Gunma University (approval no. HS2021-085).

### 4.2. Immunohistochemistry

All specimens were cut into 4 µm thick sections and mounted on glass slides. Their sections were deparaffinized with xylene, hydrated, and incubated in 0.3% hydrogen peroxide for 30 min at room temperature to block endogenous peroxidase activity. Antigen retrieval was performed in ImmunoSaver (Nisshin EM, Tokyo, Japan) at 98 °C for 45 min. Nonspecific binding sites were blocked by incubation with Protein Block Serum-Free (Dako, Carpinteria, USA) for 30 min at room temperature. The primary antibody for LPS (Hycult Biotech, PB Uden, Netherlands, HM6011, Mouse mAb, 1:100 dilution) was diluted with Dako REAL Antibody Diluent and incubated overnight at 4 °C. The Histofine Simple Stain MAX-PO (Multi) Kit (Nichirei, Tokyo, Japan), which was used as the secondary antibody, was incubated for 30 min at room temperature. The stain 3,3-diaminobenzidine tetrahydrochloride was applied as a 0.02% solution in 50 mM of ammonium acetate–citrate acid buffer (pH 6.0) containing 0.005% hydrogen peroxide. Sections were lightly contrast stained with hematoxylin and then mounted. We divided LPS into four groups: negative, weak, moderate, and strong, with moderate and strong indicating positivity. For TGFBI, CD8, and PD-L1, we employed the same staining and evaluation methods as previously reported [18]. TGFBI (Proteintech Group, Illinois, USA, Anti-TGFBI/BIGH3 antibody, 1:200 dilution), PD-L1 (Cell Signaling Technology, Massachusetts, USA, E1L3 N Rabbit mAb, 1:200 dilution), and CD8 (Abcam, Cambridge, UK, Anti-CD8, 1:500 dilution) were used. We also employed the same staining and evaluation method for wnt3a [32]. Wnt3a (Bioss Antibodies Inc, Massachusetts, USA, Wnt3a Polyclonal Antibody, 1:200 dilution) was used. We performed immunohistochemical staining using E-cadherin antibody (Takara, Otsu, Japan, HECD-1, mouse monoclonal, 1:500 dilution), with sections boiled in 10 mM citrate buffer (pH 6.0) at 98 °C for 30 min for E-cadherin activation. A positive case was defined as that in which ≥50% of the cancer cells had moderate staining intensity in E-cadherin.

### 4.3. Statistical Analysis

Statistically significant differences were analyzed using the Mann–Whitney U test for continuous variables and the Chi squared test for categorical variables. We conducted a Fisher’s exact test in Table 2 because of the small sample size. And, a *p*-value of less than 0.1 was considered a tendency. Survival rates were calculated using the Kaplan–Meier method, with statistical significance being determined by the log rank test. All analyses were performed using JMP Pro software ver 15.0 (SAS Institute Inc., Cary, NC, USA), with *p* < 0.05 indicating statistical significance.

## 5. Conclusions

The current study showed that lipopolysaccharide positivity was associated with wnt3a, TGF-β signaling, and epithelial–mesenchymal transition and was considered to tend to promote therapeutic resistance to nivolumab.

## Figures and Tables

**Table 1:** Relationship between lipopolysaccharides and clinicopathological factors.

Variable	Presence of LPS		*p*-Value
	Negative	Positive	
	*n* = 150	*n* = 48	
	No. (%)	No. (%)	
Age (years)			
	65	63	0.20
Sex			
Male	108 (72.0%)	30 (62.5%)	0.22
Female	42 (28.0%)	18 (37.5%)	
Tumor size (mm)			
	65.0 ± 2.88	63.4 ± 5.01	0.78
Depth			
m, sm, mp	41 (27.3%)	9 (18.8%)	0.22
ss, se, si	109 (72.7%)	39 (81.2%)	
Differentiation			
tub1,tub2,pap	58 (38.7%)	15 (31.3%)	0.35
por,sig	92 (61.3%)	33 (68.7%)	
Lymph node metastasis			
Absent	48 (32.0%)	15 (31.3%)	0.92
Present	102 (68.0)	33 (68.7%)	
Stage			
I II	77 (51.3%)	19 (39.6%)	0.13
III IV	71 (48.7%)	29 (60.4%)	
TGFBI in stromal			
Low expression	105 (70.0%)	16 (33.3%)	<0.0001 *
High expression	44 (30.0%)	32 (66.7%)	
CD8			
Low expression	50 (33.3%)	12 (25.0%)	0.27
High expression	100 (66.7%)	36 (75.0%)	
HER2 score			
0	103 (68.7%)	38 (79.2%)	0.012 *
1,2,3	32 (31.3%)	3 (20.8%)	
PD-L1			
Low expression	127 (84.7%)	31 (64.6%)	0.0029 *
High expression	22 (15.3%)	17 (35.4%)	
Wnt3a			
Low expression	70 (46.7%)	11 (22.9%)	0.0028 *
High expression	80 (53.3%)	37 (77.1%)	
E-cadherin			
Low expression	69 (46.0%)	33 (68.8%)	0.0055 *
High expression	81 (54.0%)	15 (31.2%)	

LPS; lipopolysaccharides, TGFBI; transforming growth factor-beta-induced protein, HER2; human epidermal growth factor receptor 2, PD-L1; Programmed cell Death ligand 1. * *p <* 0.05.

**Table 2:** Relationship between lipopolysaccharides (LPS) and nivolumab sensitivity.

Variable	Presence of LPS		*p*-Value
	Negative	Positive	
	*n* = 3	*n* = 17	
	No. (%)	No. (%)	
PR + SD	2 (66.7%)	2 (11.8%)	0.088 **
PD	1 (33.3%)	15 (88.2%)	

LPS; lipopolysaccharides, PR; partial response, SD; stable disease, PD; progressive disease. ** *p <* 0.1.

## Data Availability

The data presented in this study are available on request from the corresponding authors.
